# First Case of Zoonotic Transmission of Streptococcus equi Subspecies zooepidemicus From Cat to Human

**DOI:** 10.7759/cureus.46306

**Published:** 2023-10-01

**Authors:** Christodoulos Dolapsakis, Charalampos Charalampidis, Maria Kkirgia, Pinelopi Kollia

**Affiliations:** 1 Internal Medicine, National and Kapodistrian University of Athens School of Medicine, Attikon University Hospital, Athens, GRC; 2 Medicine, National and Kapodistrian University of Athens School of Medicine, Athens, GRC

**Keywords:** cellulitis, bacteremia, streptococcus zooepidemicus, human, cat

## Abstract

*Streptococcus equi* subspecies *zooepidemicus *is a pathogen of veterinary interest that causes disease in horses, pigs, and dogs and is recognized as an emerging cause of feline respiratory disease. Human zoonotic disease is rare but can occur in patients who are taking care of horses and via consumption of unpasteurized animal products. We describe a case of soft tissue infection and bacteremia in an elderly patient who had contact with a cat presenting respiratory symptoms and was treated with antibiotics. To the best of our knowledge, this is the first description of cat-to-human transmission of *Streptococcus zooepidemicus*.

## Introduction

*Streptococcus equis* subspecies (subsp.) *zooepidemicus *is a β-hemolytic Lancefield group C streptococcus of veterinary interest. It is found in the normal microbiota of the upper respiratory tract of horses and other animals and can also act as an opportunistic pathogen causing small-scale epidemics. Additionally, it has been recognized as a cause of human zoonotic disease in patients taking care of animals (mostly horses) or consuming unpasteurized animal products (mostly dairy products), manifesting either as bloodstream infection or localized disease. S*treptococcus zooepidemicus (S. zooepidemicus​​​​​)* is known to cause disease in cats, but cat-to-human transmission has not been described in literature before. We present a case of soft tissue infection and bacteremia due to *S. zooepidemicus* in a patient who had a minor trauma from a wandering cat with respiratory symptoms.

## Case presentation

A woman in her 90s presented to our hospital due to altered mental status and fever. Her medical history included heart failure with preserved ejection fraction, atrial fibrillation, and hypothyroidism. She lived alone in a ground-floor apartment in an urban district and did not have any domestic animals. The patient was in her usual state of health until two days before admission when, during the morning hours, a feral cat wandering in her garden bit her in the dorsal side of the right forearm while she was trying to feed her. During the next hours, erythema in the forearm at the site of the bite developed, associated with tenderness to palpation. The next day, pain and erythema worsened despite the use of paracetamol. Six hours before admission and 48 hours after the cat bite, the patient became febrile and gradually somnolent with a temperature up to 39ºC and was transferred to the emergency department of our hospital by ambulance.

At physical examination, body temperature was 39.4ºC, blood pressure 93/57 mm Hg, heart rate 130 beats per minute, and oxygen saturation 96% on ambient air. Her electrocardiogram showed atrial fibrillation without findings suggestive of ischemia. There was erythema, warmth, and edema in the dorsal surface of the right forearm without obvious traumatic lesion. The patient was obtunded, opening eyes, with a voice, and with confused speech. Her Glasgow Coma Scale was 13/15. Laboratory tests showed a white blood cell count of 19.000/μL with 17.750/μL neutrophils, creatinine 1.8 mg/dl, lactate 4.1 mmol/L, and C-reactive protein 159 mg/L (Table 1).

**Table 1:** Patient's laboratory data

Variable	Admission	Discharge	Reference range, adults
Hemoglobin (g/dl)	8.6	9.5	12-15
White blood cells (K/μl)	28.22	9.59	4-11
Neutrophils (K/μl)	25.10	6.94	2-8
Platelets (K/μl)	226	228	150-400
Urea (mg/dl)	68	29	16-48
Creatinine (mg/dl)	1.8	1.2	0.5-0.9
C-reactive protein (mg/l)	159	30	<6

Ultrasonography did not reveal septic thrombophlebitis. A working diagnosis of sepsis due to cellulitis was made. Administration of crystalloid fluids resulted in the restoration of blood pressure, and empirical antibiotic treatment with clindamycin and piperacillin-tazobactam was started. During the next 48 hours, the fever abated, renal function became normal, and level of consciousness improved. Seventy-two hours after admission, blood cultures showed growth of *Streptococcus equi *subsp. *zooepidemicus *that was sensitive to penicillin and resistant to tetracycline. At further questioning, the patient reported that the cat that bit her was obviously sick with nasal discharge. Empirical antibiotic treatment was de-escalated to ampicillin, and the patient was discharged home after seven days of antibiotic treatment.

## Discussion

Streptococci group C and G (GCS and GGS) of human origin constitute a single subspecies, *Streptococcus dysgalactiae* subsp. *equisimilis*. Various streptococci of animal origin also express the group C or G antigen. Among them* Streptococcus equi *subsp. *equi *(*S. equi*) and *Streptococcus equi* subsp. *zooepidemicus* (*S. zooepidemicus*) are the most important equine streptococci worldwide. *S. equi* is the cause of strangles, the most common infection in horses, and infects equids almost exclusively. On the contrary, *S. zooepidemicus* is a mucosal commensal not only in horses but in a variety of other species, including goats, sheep, cattle, pigs, dogs, monkeys, and cats. The veterinary importance of *S. zooepidemicus *has increased during the last few years; it is now recognized as an emerging zoonosis causing outbreaks of severe hemorrhagic pneumonia not only in horses but also in pigs and dogs [1].

Concerning cats, *Streptococcus canis (S. canis)* is the most prevalent feline species of streptococci, being part of the commensal flora and resulting in a wide spectrum of disease (pneumonia, meningitis, abscess formation) [2]. Until recently, *S. zooepidemicus* was thought to play no role in cat disease. Nevertheless, in 2010, the first outbreak of severe pneumonia in a cat shelter in Israel was described [3]. *S. zooepidemicus* was the main pathogen isolated both from the dead cats as well as the sick ones, which had purulent nasal discharge and cough. The role of cat colonies as an important risk factor for *S. zooepidemicus* transmission was further supported by an investigation of cat hoarding in the United States between 2009 and 2012; during intake examination, 55% of the cats with respiratory symptoms tested by polymerase chain reaction (PCR) were positive [4]. Finally, cases of pneumonia and meningoencephalitis in isolated domestic cats have also been described.

Although rare, *S. zooepidemicus* can cause severe disease in humans exposed to infected horses and epidemics via consumption of unpasteurized milk products. Bacteremia, meningitis, endocarditis, septic arthritis, abscess formation, and necrotizing myositis are described in sporadic cases [5]. The first outbreak of *S. zooepidemicus* bacteremia due to consumption of dairy milk products was described in 1988 [6]. In 2003, a similar outbreak occurred in Spain [7] and in 2006 in Finland [8]. In 2010, the first case of *S. zooepidemicus* transmission from dog to human was described in Ireland [9].

Meningitis seems to be the most common and serious manifestation of *S. zooepidemicus* human disease. In a review of 19 cases of meningitis, mortality was 30%, and patient age greater than 70 years was an adverse prognostic factor [10]. In a recent review of 13 sporadic cases of *S. zooepidemicus* zoonotic human infection, the median patient age was 57 years, and all patients had a history of contact with horses or ingested raw horse meat [5]. Bacteremia and septic arthritis were the most common manifestations, but there was no fatal outcome. *S. zooepidemicus* bacteremia in the context of foodborne outbreak carries significant mortality; from the 33 patients implicated in the three aforementioned outbreaks of *S. zooepidemicus* bacteremia, 12 died, and older age was again recognized as an adverse prognostic factor [6-8].

To the best of our knowledge, our case represents the first report of *S. zooepidemicus* bacteremia due to transmission from cat to human. Moreover, *S. zooepidemicus* cellulitis has never been described before. Although we were not able to perform molecular analysis in the cat in order to identify the pathogen, the timing of events and the patient’s history make zoonotic transmission certain. Taking into account the emerging role of *S. zooepidemicus* as a canine pathogen, this first description of cat-to-human transmission has important implications for public health. It is possible that cases of *S. zoopedidemicus* soft tissue infection after a cat bite are underreported; since all the strains implicated in zoonotic human disease are sensitive to penicillin, we can postulate that many *S. zooepidemicus* soft tissue infections are treated with commonly prescribed antibiotics in the primary care setting. Nevertheless, in view of the fact that some infections may lead to life-threatening invasive disease, the epidemiology of *S. zooepidemicus* in cats must be investigated at national and regional levels, particularly in rural regions and urban districts of lower socioeconomic status, where wandering cat colonies are common in order to develop preventing measures. In this sense, reporting of *S. zooepidemicus* in veterinary and medical authorities must be obligatory.

Concerning treatment, as mentioned before, all the *S. zooepidemicus* strains implicated in zoonotic human disease and reported in the literature are sensitive to penicillin, as in our case, so the latter is the treatment of choice. Duration of antibiotic treatment varies in the reported cases from seven days for uncomplicated bacteremia until eight weeks for brain abscess. In our patient, taking into account the absence of septic thrombophlebitis and persistent bacteremia, we chose to treat the patient with a seven-day antibiotic course.

## Conclusions

The veterinary importance of *S. zooepidemicus* has increased, and it is now recognized as an emerging canine and feline pathogen. The spread of *S. zooepidemicus* zoonosis to domestic animals keeps up with ever-increasing reports of human infection, which can lead to substantial morbidity and mortality. Our case represents the first report of cat-to-human transmission. *S. zooepidemicus* must be included in the differential diagnosis of soft tissue infection in persons who are in contact with cats showing signs of respiratory infection. Prompt empiric treatment with penicillin is the treatment of choice. Investigation of *S. zooepidemicus* epidemiology in cats at a national and regional level seems necessary in order to apply preventive measures and avoid a potentially serious public health problem.
